# Mini-review on initiatives to interfere with the propagation and clearance of alpha-synuclein in Parkinson’s disease

**DOI:** 10.1186/s40035-017-0104-6

**Published:** 2017-12-20

**Authors:** Daniel Kam Yin Chan, Ying Hua Xu, Luke Kar Man Chan, Nady Braidy, George D. Mellick

**Affiliations:** 1 Department of Aged Care and Rehabilitation, Bankstown Hospital, Bankstown, NSW 2200 Australia; 2 Ingham Institute, Liverpool, NSW 2170 Australia; 3 University of New South Wales, Faculty of Medicine, Sydney, Australia; 4 Faculty of Medical Science, Griffith University, Nathan, QLD Australia; 5 Griffith Institute for Drug Discovery, Griffith University, Nathan, QLD Australia

**Keywords:** Parkinson’s disease, α-synuclein, Endocytosis, Aggregation

## Abstract

In this mini-review, we summarize recent findings relating to the prion-like propagation of α-synuclein (α-syn) and the development of novel therapeutic strategies to target synucleinopathy in Parkinson’s disease (PD). We link the Braak’s staging hypothesis of PD with the recent evidence from in-vivo and in-vitro studies for the prion-like cell-to-cell propagation of α-syn (via exocytosis and endocytosis). The classical accumulation of aggregated α-syn in PD may result from an increased production or a failure in the mechanisms of clearance of α-syn. We discuss novel agents, currently in clinical trial for PD including the ones that impact the aggregation of α-syn and others that interfere with α-syn endocytosis as a means to target the progression of the disease.

## Background

Parkinson’s disease (PD) is a complex neurodegenerative disorder characterized by cardinal features of resting tremor, bradykinesia, rigidity and loss of balance. Clinically, PD progresses from a disability that mainly affects movement to a disabling disease that may affect memory, executive function, autonomic nervous system or other neuropsychiatric functions. Pathologically, PD is characterized by loss of dopaminergic neurons in the substantia nigra, pars compacta, and the presence of intraneuronal protein inclusions known as Lewy bodies (LBs) and Lewy neurites (LNs), and characterized collectively as Lewy pathology (LP). The principal component of the LPs contain abnormal aggregates of α-synuclein (α-syn).

In 2003, using the presence of LP as a marker of pathology, Braak [1] proposed a new pathological staging of PD which described a pre-clinical phase that commences in the olfactory and/or gastrointestinal nervous system with LP only present in the substantia nigra of the central nervous system at a later stage (or clinical phase) of the disease. This staging mirrors the clinical prodromal loss of smell and/or constipation, both of which can pre-date the classical cardinal manifestations of PD (resting tremor, bradykinesia, rigidity and loss of balance). Furthermore, increasing evidence [2] has supported the hypothesis that the propagation of α-syn fibrils forms the pathological basis of the staging phenomenon as proposed by Braak in PD. Herein, we review the recent findings on the aggregation and propagation of α-syn fibrils in PD and discuss novel therapeutic strategies which are based on the propagation hypothesis.

### α-syn and Parkinson’s disease

Human α-syn is a 140 amino acid protein expressed mostly in the brain, predominantly in neurons, albeit to a lesser extent in glial cells. It makes up 1% of protein content in neurons especially in pre-synaptic terminals. While the exact role of α-syn under normal physiological conditions is not entirely clear, studies suggest that it plays a role in maintaining a supply of synaptic vesicles in presynaptic areas [3].

Missense mutations in *SNCA* (the gene coding for α-syn) leading to A53T, A30P and E46K protein changes [4] as well as duplication, and triplication of *SNCA*, all lead to misfolding or excessive production of α-syn protein. Other genetic mutations such as PARK-*LRRK2* and PARK- *VPS35* are also associated with α-syn pathology. Aggregated α-syn is also found in other synucleinopathies e.g. Lewy body dementia, multiple system atrophy and some Alzheimer’s disease cases [5].

Although α-syn oligomers are thought to be the toxic species, it is the abnormal aggregation of α-syn into fibrils that underpins the characteristic LP seen microscopically. It is now believed that α-syn can form small aggregates and fibrils in neurons. The fibrils are formed when multiple α-syn peptides are aggregated together, with misfolding of these peptides being the triggering events, accompanied by hyperphosphorylation predominantly at serine residue 129, and to a lesser extent at serine 87 and some tyrosine residues [6]. The lack of clearance by the autophagosome-lysosome and proteasome systems are thought to be other important factors leading to oligomerization of α-syn (protofibrils) which eventually undergo aggregation to become α-syn fibrils, the major constituents in LBs or LNs [7, 8].

### The endocytosis and exocytosis hypothesis

The uptake of various forms of extracellular α-syn, including fibrils, oligomers, and monomers, into neuronal cells and their subsequent degradation are different. The uptake of α-syn fibrils could be inhibited by low temperature or the expression of a dominant-negative mutant dynamin-1 K44A, supporting the notion that the process is mediated by endocytosis. The internalized fibrils are trafficked via the endosomal pathway and are degraded in the lysosome. Non-fibrillar oligomeric α-syn aggregates are also internalized via endocytosis and degraded by the lysosome. In contrast, the internalization of monomeric α-syn is not affected by cold temperature or the expression of dynamin-1 K44A, consistent with direct translocation process across the plasma membrane. Internalized monomers rapidly pass the plasma membrane, exiting the cells before being degraded by the cellular proteolytic systems [9].

### Evidence of prion-like propagation of α-syn in Parkinson’s disease

#### Human studies

A seminal piece of evidence came from post-mortem histopathological finding that 3 out of 4 PD patients who died 11–16 years after transplantation of embryonic mesencephalic neurons had exhibited LBs and LNs in grafted neurons in contrast to 4 patients who survived only 4–9 years following transplantation who did not show the pathology [10, 11]. These clinical cases imply that the pathological prion-like propagation changes may occur albeit slowly.

However, some critics of the α-syn propagation hypothesis suggest that LP (LBs or LNs) can also be found in 8–17% of elderly aged 60 and over and is therefore an “incidental” finding [12–14]. The argument against this line of thought is that the majority of elderly at autopsy do not have “incidental” Lewy pathology. Furthermore, even mild accumulation of LBs or LNs is associated with reduced striatal tyrosine hydroxylase levels (an enzyme for l-tyrosine to DOPA) [15]. Therefore these “incidental” lesions may represent the pre-clinical prodromal phase of PD.

#### Animal studies

Lending support to the human studies are animal models studies. Luk et al. [16] demonstrated that intracellular inclusion formation in α-syn over-expressing cells can be initiated by the presence of fibrillar α-syn seedings. C57BL/6 J mice injected with α-syn fibrils developed abundant Lewy body/Lewy neurite-like pathology, whereas mice injected with soluble α-syn did not [17].

In-vivo studies showed that intracerebral injection of synthetic α-syn fibrils and/or insoluble α-syn from diseased brain changes normal α-syn into an abnormal form, and the abnormal α-syn propagates throughout the brain in a prion-like manner in wild-type mouse [16–19] and α-syn transgenic mouse [20–23]. Injection of Lewy body extracts from PD brains triggered α-syn pathology and neurodegeneration in monkeys [22]. Injection of synthetic α-syn fibrils into brains of non-transgenic primates induced PD-like α-syn pathologies within only 3 months after injection [24]. Tarutani et al. used various forms of α-syn protein and examined their seeding properties in-vitro, in cells and in mouse experimental models and found fragmented amyloid-like aggregates of short α-syn fibrils are the key pathogenic seeds that trigger prion-like conversion [19]. Bernis et al. showed that human wild-type α-syn supports the prion-like spreading of α-syn pathology in the absence of endogenously expressed mouse α-syn in-vivo [25]. A substantial review of above prion-like propagation in animal models concluded that despite discrepancies in details of the experimental paradigms and outcomes, in-vivo studies from several independent groups along with numerous in-vitro cell culture studies support the concept of cell-to-cell spread of α-syn pathology [26].

### New agents that interfere with α-syn uptake by neurons or its aggregation or enhance its clearance

#### Interfering with α-syn propagation via uptake/endocytosis

The endocytosis process of α-syn can be interfered with and it was previously shown that β-Amyloid_1–40,_ but not β-Amyloid_1–42_, interfered with the uptake of α-syn pre-formed fibrils [27]. The reduction of uptake is up to 80% compared to controls in human neurons. It was then hypothesized by the authors that the mechanism of uptake of α-syn pre-formed fibrils is probably via a “receptor mediator endocytosis” process [27]. The α-syn receptor, lymphocyte-activation gene 3 (LAG3), as identified by Mao et al. supports this hypothesis [28].

#### Interfering with α-syn aggregation

A study reported [29] that mannitol, a blood-brain barrier disrupter, can inhibit α-syn aggregation into fibrils with low concentration in-vitro. This study also revealed that mannitol had effect on PD fly and mouse models in-vivo. Mannitol dramatically corrected the behavioural defects and this was accompanied by 70% reduction in α-syn aggregates in the brains of mannitol-treated flies as compared with the untreated group. Daily intraperitoneal injections of mannitol (1 g/kg) reduced α-syn accumulation in the hippocampus, basal ganglia, and substantial nigra but not in the neocortex in the mThy1 α-syn transgenic mouse model. These results led to the hypothesis that mannitol administration in combination with other drugs could be a promising new approach for treating PD and other brain-related diseases such as Alzheimer’s disease. However, more in-vivo experiments, including behaviour testing, will need to be carried out before any testing with humans can be attempted.

#### Immunotherapy

Antibodies against α-syn have shown therapeutic effects in mouse models of synucleinopathy and have reduced pathological and behavioural changes of those mice. Immunization against α-syn resulted in reduced α-syn accumulation and synaptic loss in a transgenic mouse model and antibodies against α-syn assisted in the clearance of extracellular α-syn proteins by microglia, thereby preventing their actions on neighboring cells [30]. Games [31] and Spencer [32] both found in the mThy1-α-syn transgenic mouse model resembling the striato-nigral and motor deficits of PD, monoclonal antibodies directed against the C-terminus of α-syn attenuated synaptic and axonal pathology, reduced the accumulation of C-terminus-truncated α-syn in axons, rescued the loss of tyrosine hydroxylase fibres in striatum, and improved motor and memory deficits.

Lindstrom et al. [33] have shown intraperitoneal injections of mAb47 into 14 month old Thy-1 transgenic mice given weekly for 14 weeks have resulted in significantly lower levels of soluble membrane-associated protofibrils in spinal cord accompanied by a reduction of motor dysfunction. Tran et al. [34] demonstrated that in-vitro monoclonal antibodies against α-syn could prevent α-syn pre-formed fibrils uptake and cell-to-cell transmission, therefore reducing α-syn pre-formed fibrils-induced LB/LN formation and rescue synapse/neuron. They also showed that intraperitoneal injection of monoclonal antibodies specific for misfolded α-syn into wild-type mice injected intrastriatally with α-syn pre-formed fibrils could reduce LB/LN pathology, ameliorate substantia nigra dopaminergic neuron loss, and motor dysfunction.

Using AFFITOPE (AFF I) vaccine, Mandler and Valera have demonstrated vaccination against mThy-1- α-syn transgenic Parkinson’s disease mice model was effective in eliciting a high antibody response in CSF and plasma with the antibody capable of crossing blood-brain-barrier [35]. The treatment reduces accumulation of α-syn oligomers in neural tissues, subsequently reducing neurodegeneration and improvement in clinical deficits. Similarly, they also demonstrated AFF 1 was effective against transgenic Multiple-system Atrophy mice [35].

Neuropore has developed a tailor-made peptide vaccine NPT200–11, a drug that can prevent the aggregation of α-syn. Using this discovery as basis, AFFiRiS vaccine trial has currently begun in humans [36]. Phase1 trial showed that it was (4 vaccine injections) safe, and 19 out of 22 participants developed antibodies against α-syn. In phase 2 trial over a 3 year observation, 8 out of 19 participants did not require increase in L-dopa therapy; and 5 out of 8 had no deterioration of the motor score.

Other clinical trials using monoclonal antibody targeting aggregated α-syn are also underway. Biogen with BIIB-054 [37] and Prothena/Roche with PRX002/RO7046015 [38, 39] have both completed phase 1 studies with promising safety results and are heading toward phase 2 trials. PRX002/RO7046015 was shown to be effective in CNS penetration and significant reduction of free serum α-syn in a double-blind, placebo-controlled trial involving 80 patients with PD.

#### Increasing the degradation of α-syn in cells via the autophagy-lysosome system

The therapeutic potential of upstream macroautophagy-enhancing agents (e.g. rapamycin and lithium) may be limited due to their lack of selectivity and the double-edged sword properties of macroautophagy. Recently developed compounds that selectively target downstream components of the autophagy-lysosomal pathway (including Transcription Factor EB, lysosomes, GCase), as well as chaperone-mediated autophagy regulators, may exert more specific effects on autophagy and may have better therapeutic perspective [40].

Mak et al. found when neuronal α-syn expression is increased either as a result of toxic injury or transgenic overexpression in mice, there is marked concomitant elevation of lysosome-associated membrane protein type 2A (LAMP-2A) [8]. Others have further found overexpression of LAMP-2A leads to upregulation of chaperone-mediated autophagy in human neuroblastoma SH-SY5Y cells, rat primary cortical neurons in-vitro, and nigral dopaminergic neurons in-vivo [41]. Hence, induction of chaperone-mediated autophagy may be a novel therapeutic option in the clearance of α-syn.

Indeed two herbal medications have been found to be potential candidates of enhancing autophagy. Isorhynchophylline is a natural alkaloid which has been shown to promote clearance of wild-type, A53T and A30P α-syn aggregations in neuronal cells including dopaminergic neurons via the autophagy-lysosome pathway [42]. Likewise, Paeoniflorin, a major active ingredient of a traditional herbal medication Radix, has been found to play a neuroprotective role by up-regulating autophagy and the ubiquitin-proteasome degradation pathway [43].

There is also preliminary evidence that poly(DL-lactide-co-glycolide) (PLGA) acidic nanoparticles (aNP) restore impaired lysosomal function in a series of toxin and genetic cellular models of PD, adding to the range of therapeutic options of promoting the degradation of α-syn [44].

## Conclusion

There is an increasing body of evidence to support the notion that α-syn propagate by endocytosis and exocytosis. This spread, induction of further α-syn synthesis, failure of clearance, aggregation and formation of fibrils and cell death form the basis in elucidating how PD progresses clinically.

Traditional treatment for PD is based on dopamine supplement. Treatment options currently available would only alleviate symptoms for a while (and this includes deep brain stimulation and duodopa). If the α-syn hypothesis is true, for the first time, there is a possibility of modifying the course of illness provided the anti- α-syn therapies work as suggested. Finally, the cautionary tale of the β-amyloid story for Alzheimer’s disease and failed novel therapies based on this hypothesis should remind us to be cautious of our hope. While laboratory and animal studies can be promising, when applied to humans, the result may be disappointing. Therefore, it is early days and we eagerly look forward to the trial results.

## Abbreviations

a-syn: a-synuclein
LAG3: lymphocyte-activation gene 3
LB: Lewy bodies
LN: Lewy neurites
LP: Lewy pathology
PD: Parkinson’s disease
